# Racial and Ethnic Disparities Among COVID-19 Cases in Workplace Outbreaks by Industry Sector — Utah, March 6–June 5, 2020

**DOI:** 10.15585/mmwr.mm6933e3

**Published:** 2020-08-21

**Authors:** David P. Bui, Keegan McCaffrey, Michael Friedrichs, Nathan LaCross, Nathaniel M. Lewis, Kylie Sage, Bree Barbeau, Dede Vilven, Carolyn Rose, Sara Braby, Sarah Willardson, Amy Carter, Christopher Smoot, Andrea Winquist, Angela Dunn

**Affiliations:** ^1^Epidemic Intelligence Service, CDC; ^2^Division of Environmental Health Science and Practice, National Center for Environmental Health, CDC; ^3^Utah Department of Health, Salt Lake City, Utah; ^4^Salt Lake County Health Department, Salt Lake City, Utah; ^5^Summit County Health Department, Park City, Utah; ^6^Southeast Utah Health Department, Price, Utah; ^7^Davis County Health Department, Clearfield, Utah; ^8^Weber-Morgan Health Department, Ogden, Utah; ^9^Wasatch County Health Department, Heber, Utah.

*On August 17, 2020, this report was posted online as an *MMWR *Early Release.*

Improved understanding of the overall distribution of workplace coronavirus disease 2019 (COVID-19) outbreaks by industry sector could help direct targeted public health action; however, this has not been described. The Utah Department of Health (UDOH) analyzed COVID-19 surveillance data to describe workplace outbreaks by industry sectors. In this report, workplaces refer to non–health care, noncongregate–living, and noneducational settings. As of June 5, 2020, UDOH reported 277 COVID-19 outbreaks, 210 (76%) of which occurred in workplaces. Approximately 12% (1,389 of 11,448) of confirmed COVID-19 cases in Utah were associated with workplace outbreaks. The 210 workplace outbreaks occurred in 15 of 20 industry sectors;* nearly one half of all workplace outbreaks occurred in three sectors: Manufacturing (43; 20%), Construction (32; 15%) and Wholesale Trade (29; 14%); 58% (806 of 1,389) of workplace outbreak-associated cases occurred in these three sectors. Although 24% of Utah’s workforce in all 15 affected sectors identified as Hispanic or Latino (Hispanic) or a race other than non-Hispanic white (nonwhite^†^) (*1*), 73% (970 of 1,335) of workplace outbreak-associated COVID-19 cases were in persons who identified as Hispanic or nonwhite. Systemic social inequities have resulted in the overrepresentation of Hispanic and nonwhite workers in frontline occupations where exposure to SARS-CoV-2, the virus that causes COVID-19, might be higher (*2*); extra vigilance in these sectors is needed to ensure prevention and mitigation strategies are applied equitably and effectively to workers of racial and ethnic groups disproportionately affected by COVID-19. Health departments can adapt workplace guidance to each industry sector affected by COVID-19 to account for different production processes and working conditions.

Data on workplace COVID-19 outbreaks occurring during March 6–June 5, 2020, were collected from UDOH’s COVID-19 case surveillance system. UDOH defined workplace outbreaks as the occurrence of two or more laboratory-confirmed COVID-19 cases occurring within the same 14-day period among coworkers in a common workplace (i.e., same facility). UDOH classifies outbreaks in congregate living facilities, educational institutions, and health care facilities as distinct outbreak types that are managed differently from general workplace outbreaks because of the special populations they serve and the setting-specific guidance they require. Thus, cases from these settings were not included in this analysis of workplace outbreaks. Case investigators collected facility addresses, business names, or both for all workplace outbreaks. Workplaces were classified according to the North American Industry Classification System (NAICS; https://www.census.gov/eos/www/naics/) into one of 20 industry sectors. NAICS codes for workplaces were obtained from Utah’s Division of Corporations and Commercial Code directory of registered businesses (https://secure.utah.gov/bes/). Because of small case numbers and similarities in sector processes and settings, the sectors for Professional, Scientific, and Technical services and Information were combined into a single category, as were the Finance and Insurance, Real Estate and Rental and Leasing, and Public Administration sectors.

The distribution of workplace outbreaks and associated cases across sectors was described. Outbreak incidence (cases per 100,000 workers) was calculated using Utah sector workforce estimates reported in the 2019 Census Quarterly Workforce Indicators (*1*) for sector denominators; workforce estimates were not adjusted to remove workers affected by outbreaks in excluded settings (e.g., educational workers and health care workers). Descriptive statistics and chi-squared tests were used to summarize and compare demographics and outcomes (e.g., hospitalization) of persons with workplace outbreak-associated COVID-19 with persons of working age (≥15 years) with nonoutbreak–associated COVID-19 (i.e., cases not associated with an outbreak). To identify sectors in which COVID-19 racial and ethnic disparities might be unrecognized, the racial and ethnic composition of workplace outbreak-associated cases were compared with the overall racial and ethnic composition in each sector in Utah. All statistical analyses were done in R (version 3.6.1; The R Foundation) p-values <0.05 were considered statistically significant.

During March 6–June 5, 2020, UDOH reported 11,448 confirmed COVID-19 cases throughout Utah, including 1,389 (12%) associated with workplace outbreaks, 1,081 (9%) associated with outbreaks in other settings (i.e., congregate living, educational, health care), and 8,978 (78%) that were not associated with an outbreak. UDOH reported 210 workplace COVID-19 outbreaks (median cases per workplace outbreak = 4; range = 2–79) involving 15 industry sectors, most frequently in Manufacturing (43; 20%), Construction (32; 15%), and Wholesale Trade (29; 14%); these three sectors accounted for 58% (806 of 1,389) of workplace outbreak-associated cases (Table 1). The incidence among workplace outbreak-associated cases was highest in the Wholesale Trade (377 per 100,000 workers) and Manufacturing (339 per 100,000 workers) sectors.

**TABLE 1:** Distribution of workplace outbreaks and workplace-associated COVID-19 cases, by North American Industry Classification System (NAICS) industry sector, and demographic characteristics of persons with workplace-associated COVID-19 and their outcomes – Utah, March 6–June 5, 2020

NAICS industry sector code	Industry sector	Workers, outbreaks, and cases no. (%)			Workplace outbreak-associated incidence^†^	Characteristic no. (%)		
		Workforce*	Workplace outbreaks	Workplace outbreak-associated cases		Hispanic or nonwhite^§^	Admitted to hospital^¶^	Severe outcomes^¶^
**Overall total**	**—**	**1,305,130 (100)**	**210 (100)**	**1,389 (100)**	**106.4**	**970/1,335 (73)**	**85/1,382 (6)**	**40/1,155 (3)**
31–33	Manufacturing	137,579 (11)	43 (20)	467 (34)	339.4	365/444 (82)	25/464 (5)	12/464 (3)
42	Wholesale Trade	53,045 (4)	29 (14)	200 (14)	377.0	145/190 (76)	8/197 (4)	3/197 (2)
23	Construction	113,610 (9)	32 (15)	139 (10)	122.3	97/135 (72)	11/139 (8)	7/139 (5)
44, 45	Retail Trade	169,559 (13)	28 (13)	116 (8)	68.4	78/113 (69)	5/116 (4)	1/116 (1)
56	Administrative, Support, and Waste Management	95,878 (7)	9 (4)	114 (8)	118.9	68/109 (62)	8/114 (7)	2/114 (2)
72	Accommodation and Food Services	128,983 (10)	25 (12)	100 (7)	77.5	78/97 (80)	7/100 (7)	7/100 (7)
48, 49	Transportation and Warehousing	64,360 (5)	10 (5)	97 (7)	150.7	71/94 (76)	9/97 (9)	6/97 (6)
71	Arts, Entertainment, and Recreation	34,862 (3)	6 (3)	40 (3)	114.7	14/39 (36)	2/40 (5)	0/40 (0)
51, 54	Professional, Scientific, Technical, and Information**	151,275 (12)	9 (4)	47 (3)	31.1	20/46 (43)	5/47 (11)	2/47 (4)
52, 53, 92	Finance, Real Estate, and Public Administration**	147,220 (11)	6 (3)	24 (2)	16.3	10/24 (42)	1/23 (4)	0/23 (0)
81	Other Services (except Public Administration)	38,651 (3)	8 (4)	24 (2)	62.1	13/23 (57)	3/24 (13)	1/24 (4)
62	Health Care and Social Assistance^††^	170,108 (13)	5 (2)	21 (2)	12.3	11/21 (52)	1/21 (5)	0/21 (0)

**Abbreviation:** COVID 19 = coronavirus disease 2019.

* Based on U.S. Census Quarterly Workforce Indicators, Utah 2019 (third quarter). https://qwiexplorer.ces.census.gov/static/explore.html#x=0&g=0.

^†^ Cases per 100,000 workers. Estimated as workplace outbreak-associated COVID-19 cases per 100,000 workers in industry sector; does not include cases among workers not part of a workplace outbreak.

^§^ Among cases with known race and ethnicity (n = 1,335); Hispanic includes Hispanic or Latino; nonwhite includes the following (all non-Hispanic): black or African American, American Indian or Alaska Native, Asian, Native Hawaiian or Other Pacific Islander, two or more races, or other race groups.

^¶^ Among cases with known hospitalization (n = 1,382) or severity status (n = 1,155); severe outcome defined as intensive care unit admission, mechanical ventilation, or death.

** Because of small case numbers, Information (NAICS code 51) and Professional, Scientific, and Technical services (NAICS code 54) sectors were combined into a single category; Finance and Insurance (NAICS code 52), Real Estate and Rental and Leasing (NAICS code 53), and Public Administration (NAICS code 92) sectors were also combined into a single category.

^††^ The full name of this NAICS sector includes “Health Care”; however, because health care settings were not included in this analysis, they represent only social assistance businesses.

Compared with persons aged ≥15 years with nonoutbreak–associated COVID-19 (median age = 38 years), persons with workplace outbreak-associated COVID-19 were older (median age = 41 years) (Mann-Whitney test, p = 0.01), more likely to identify as Hispanic (56.4% versus 39.8%; p <0.001), and more likely to be male (61.4% versus 50.6%; p <0.001) (Table 2). The proportion of patients hospitalized was significantly lower among persons with workplace outbreak-associated COVID-19 (6.1%) than among those with nonoutbreak–associated COVID-19 (7.6%) (p = 0.01).

**TABLE 2:** Characteristics of nonoutbreak–associated cases and workplace outbreak-associated cases of COVID-19 among persons aged ≥15 years — Utah, March 6–June 5, 2020.

Characteristic	Case status no. (%)		P-value*
	Not outbreak–associated	Workplace outbreak–associated	
	(n = 8,297)	(n = 1,389)	
**Age group, yrs**			<0.001
15–24	1,718 (20.7)	192 (13.8)	
25–44	3,489 (42.1)	658 (47.4)	
45–64	2,360 (28.4)	493 (35.5)	
≥65	730 (8.8)	46 (3.3)	
**Race/Ethnicity**			<0.001
Hispanic or Latino	3,303 (39.8)	783 (56.4)	
White, non-Hispanic	2,972 (35.8)	365 (26.3)	
Native Hawaiian or Pacific Islander, non-Hispanic	317 (3.8)	61 (4.4)	
Asian, non-Hispanic	194 (2.3)	42 (3.0)	
Black or African American, non-Hispanic	247 (3.0)	38 (2.7)	
American Indian or Alaska Native, non-Hispanic	309 (3.7)	13 (0.9)	
Other, non-Hispanic	237 (2.9)	33 (2.4)	
Missing	718 (8.7)	54 (3.9)	
**Ethnicity**			<0.001
Non-Hispanic	4,279 (51.6)	552 (39.7)	
Hispanic	3,303 (39.8)	783 (56.4)	
Missing	715 (8.6)	54 (3.9)	
**Sex**			<0.001
Female	4,088 (49.3)	536 (38.6)	
Male	4,199 (50.6)	853 (61.4)	
Missing	10 (0.1)	0 (0)	
**Any chronic condition**			0.24
Yes	2013 (24.3)	318 (22.9)	
No	1698 (20.5)	298 (21.5)	
Missing	4586 (55.3)	773 (55.7)	
**Hospitalized**			0.01
Yes	630 (7.6)	85 (6.1)	
No	7,136 (86.0)	1,297 (93.4)	
Missing	531(6.4)	7 (0.5)	
**Severe outcome†**			0.74
Yes	217 (2.6)	40 (2.9)	
No	5,618 (67.7)	1,115 (80.3)	
Missing	2,462 (29.7)	234 (16.8)	
**ICU admission**			0.94
Yes	195 (2.4)	36 (2.6)	
No	7,497 (90.4)	1,341 (96.5)	
Missing	605 (7.3)	12 (0.9)	
**Mechanical ventilation**			0.78
Yes	84 (1.0)	14 (1.0)	
No	7,111 (85.7)	1,339 (96.4)	
Missing	1,102 (13.3)	36 (2.6)	
**Died**			0.61
Yes	59 (0.7)	9 (0.6)	
No	5,947 (71.7)	1,153 (83.0)	
Missing	2,291 (27.6)	227 (16.3)	

**Abbreviations:** COVID-19 = coronavirus disease 2019; ICU = intensive care unit.

* P-values based on chi-squared tests and excludes missing categories; level of significance = p<0.05.

^†^ Persons with COVID-19 were classified as having a severe outcome if they were admitted to an ICU, required mechanical ventilation, or died; they were classified as not having a severe outcome if they were not admitted to an ICU, did not require mechanical ventilation, and did not die.

Among persons with workplace outbreak-associated COVID-19, information on race and ethnicity was available for 1,335 (96%); 783 (59%) workers with workplace outbreak-associated COVID-19 identified as Hispanic, 365 (27%) as non-Hispanic white, and 187 (19%) as nonwhite. In total, 970 (73%) of persons with workplace outbreak-associated COVID-19 identified as Hispanic or nonwhite, although these ethnic/racial groups represent <24% of Utah’s workforce in the 15 affected industry sectors (*1*). This disparity was observed across all 15 industry sectors with the largest in Wholesale Trade (percentage point difference between percentage of Hispanic or nonwhite workers among workplace outbreak-associated COVID-19 cases and the overall workforce = 58) and Manufacturing (percentage point difference = 53) sectors (Figure).

**FIGURE:**
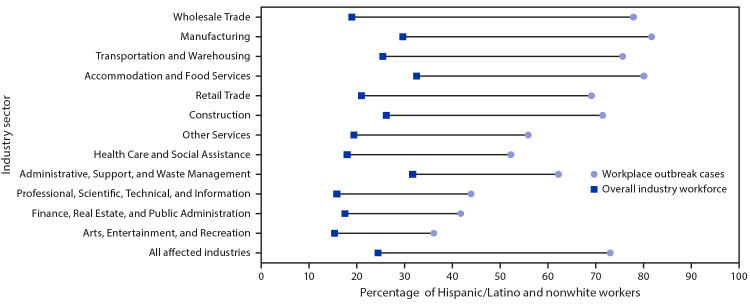
Percentage point difference* between the percentage of workers with workplace outbreak-associated COVID-19 who are Hispanic/Latino and nonwhite† and the percentage of Hispanic/Latino and nonwhite workers within the entire industry workforce,^§^ by industry sector^¶^ — Utah, March 6–June 5, 2020 **Abbreviation:** COVID-19 = coronavirus disease 2019. * Sectors are sorted on absolute disparity between the percentage of Hispanic/Latino and nonwhite workers among workplace outbreak cases and the percentage of Hispanic/Latino and nonwhite workers in the overall industry workforce, in descending order. ^†^ Nonwhite includes the following (all non-Hispanic): black or African American, American Indian or Alaska Native, Asian, Native Hawaiian or Other Pacific Islander, two or more races, or other race groups. ^§^ Sector workforce demographics from U.S. Census Quarterly Workforce Indicators, Utah 2019 (third quarter); https://qwiexplorer.ces.census.gov/static/explore.html. ^¶^ Industry sectors are based on the North American Industry Classification System (https://www.census.gov/eos/www/naics/). Because of small case numbers and similarities in sector processes and settings, Professional, Scientific, and Technical Services and Information sectors were combined into a single category, as were Finance and Insurance, Real Estate, Rental and Leasing, and Public Administration.

## Discussion

During March 6–June 5, COVID-19 outbreaks were identified in nearly all assessed industry sectors in Utah, with approximately one half of workplace outbreak-associated cases occurring in three sectors: Manufacturing, Construction, and Wholesale Trade. Persons with workplace outbreak-associated COVID-19 were disproportionately Hispanic or nonwhite compared with overall racial/ethnic distributions in these industry sectors. Sector-specific COVID-19 guidance, which CDC has generated for many industries,^§^^,^^¶^^,^** should be followed to account for different production processes, business operations, and working conditions faced by workers in these sectors. When available, efforts should be made to help employers operationalize sector-specific guidance; CDC and UDOH plain-language business guides can help employers manage and prevent workplace outbreaks and exposures.^††^ Avoiding introduction of SARS-CoV-2 into workplaces is critical to preventing outbreaks, making both community- and workplace-specific interventions important if SARS-CoV-2 transmission in workplace settings is to be prevented. Health departments and employers need to ensure mitigation strategies are provided using culturally and linguistically responsive materials and messages, which reach workers of racial and ethnic minority groups, especially those disproportionately affected by workplace COVID-19 outbreaks.

The racial and ethnic disparities in workplace outbreak-associated COVID-19 cases found in Utah and identified in meat processing facility outbreaks in other states (*3*) demonstrate a disproportionate risk for COVID-19. These disparities might be driven, in part, by longstanding health and social inequities (*2*), resulting in the overrepresentation of Hispanic and nonwhite workers in frontline occupations (i.e., essential and direct-service) where risk for SARS-CoV-2 exposure might be higher than that associated with remote or nondirect–service work (*4*). In addition, Hispanic and nonwhite workers have less flexible work schedules and fewer telework options compared with white and non-Hispanic workers (*5*). Lack of job flexibility (i.e., ability to vary when to start and end work), lack of telework options, and unpaid or punitive sick leave policies might prevent workers from staying home and seeking care when ill, resulting in more workplace exposures, delayed treatment, and more severe COVID-19 outcomes (*6*,*7*). Whenever employers can provide flexible work schedules, nonpunitive paid sick leave, and telework options, they should offer this equitably to Hispanic and nonwhite workers.

The findings in this report are subject to at least six limitations. First, this analysis is not representative of all workplace outbreaks in Utah. Outbreaks might not be detected or reported in smaller workplaces, and workers with self-limiting symptoms might not be tested. Outbreaks in nursing homes, detention centers, and education settings were not included in this analysis, and thus, the relative impact of COVID-19 in industry sectors represented by those workers were not assessed. Second, worker-to-worker transmission could not be confirmed; some workplace outbreak-associated cases will represent community and household transmission, or transmission between coworkers outside of work (e.g., commuting to work or social gatherings). Third, individual occupation data were unavailable, so assumptions about the types of affected workers (e.g., frontline workers) cannot be confirmed. Gathering detailed individual occupation data during case investigations might help inform more targeted risk-mitigation interventions within sectors by identifying types of work and workers at highest risk for SARS-CoV-2 infection. Fourth, the stay-at-home directives in effect in Utah during the study period likely differentially affected workplace attendance in different sectors (e.g., more telework in information than in construction sectors); therefore, these findings might not be generalizable to states with different restriction levels and sector workforce distributions. Fifth, it is not known to what extent workers in these sectors were familiar with, able, and willing to follow guidance to prevent and reduce the spread of SARS-CoV-2. Finally, workforce estimates used to calculate the outbreak incidence rates by sector could not be adjusted to account for workers in health care, educational, and congregate-living settings that were excluded from this analysis, resulting in underestimated rates; outbreak incidence rates for the Educational Services sector (NAICS code 61) and Health Care and Social Services sector (NAICS code 62) were likely most affected by this limitation.

Understanding the distribution of workplace outbreaks across industry sectors can help health departments identify and target industries where additional guidance and intervention to mitigate SARS-CoV-2 transmission might be needed. Further, health departments should consider obtaining case occupation data to better understand workplace outbreaks to inform more targeted interventions. The overrepresentation of Hispanic and nonwhite workers in frontline occupations has resulted in disproportionate disease incidence among racial/ethnic minority groups. Care must be taken to ensure that prevention and mitigation strategies are applied equitably and effectively using culturally and linguistically responsive materials, media, and messages to workers of racial and ethnic minority groups disproportionately affected by COVID-19.

**Summary**

**What is already known about this topic?**

COVID-19 outbreaks occur within various workplaces.

**What is added by this report?**

During March 6–June 5, 2020, workplace outbreaks occurred in 15 Utah industry sectors; 58% of workplace outbreak-associated COVID-19 cases were in three sectors: Manufacturing, Wholesale Trade, and Construction. Despite representing 24% of Utah workers in all affected sectors, Hispanic and nonwhite workers accounted for 73% of workplace outbreak-associated COVID-19 cases.

**What are the implications for public health practice?**

Sector-specific COVID-19 guidance should be followed. Mitigation strategies should be culturally and linguistically responsive to racial/ethnic minority workers disproportionately affected by COVID-19. Collection of detailed case occupation data is needed to understand types of work where exposure risk is highest.

## Footnotes

* The 20 industry sectors include Agriculture, Forestry, Fishing and Hunting; Mining, Quarrying, and Oil and Gas Extraction; Utilities; Construction; Manufacturing; Wholesale Trade; Retail Trade; Transportation and Warehousing; Information; Finance and Insurance; Real Estate and Rental and Leasing; Professional, Scientific, and Technical Services; Management of Companies and Enterprises; Administrative and Support and Waste Management and Remediation Services; Educational Services; Health Care and Social Assistance; Arts, Entertainment, and Recreation; Accommodation and Food Services; Other Services (except Public Administration); and Public Administration (https://www.census.gov/eos/www/naics/). No workplace outbreaks were reported in the following sectors: Agriculture, Forestry, Fishing and Hunting; Mining, Quarrying, and Oil and Gas Extraction; Utilities; Management of Companies and Enterprises; and Educational Services.

† Nonwhite includes the following (all non-Hispanic): black or African American, American Indian or Alaska Native, Asian, Native Hawaiian or Other Pacific Islander, two or more races, or other race groups.

§ https://www.cdc.gov/coronavirus/2019-ncov/community/organizations/businesses-employers.html; https://www.cdc.gov/niosh/emres/2019_ncov.html.

¶ https://www.cdc.gov/coronavirus/2019-ncov/community/organizations/construction-workers.html.

** https://www.cdc.gov/coronavirus/2019-ncov/community/guidance-manufacturing-workers-employers.html.

†† https://coronavirus.utah.gov/business/; https://www.cdc.gov/coronavirus/2019-ncov/community/guidance-business-response.html.

## References

[1] US Census Bureau. QWI Explorer. Suitland, MD: US Department of Commerce, US Census Bureau; 2020. https://qwiexplorer.ces.census.gov/static/explore.html#x=0&g=0

[2] Thakur N, Lovinsky-Desir S, Bime C, Wisnivesky JP, Celedón JC; Health Equality and Diversity Committee of the American Thoracic Society. The structural and social determinants of the racial/ethnic disparities in the U.S. COVID-19 pandemic: what’s our role? Am J Respir Crit Care Med 2020. Epub July 17, 2020. 10.1164/rccm.202005-1523PP 32677842 PMC7528789

[3] Waltenburg MA, Victoroff T, Rose CE, ; COVID-19 Response Team. Update: COVID-19 among workers in meat and poultry processing facilities—United States, April–May 2020. MMWR Morb Mortal Wkly Rep 2020;69:887–92. 10.15585/mmwr.mm6927e2 32644986 PMC7732361

[4] Rho HJ, Brown H, Fremstad S; Center for Economic and Policy Research. A basic demographic profile of workers in frontline industries. Washington, DC: Center for Economic and Policy Research; 2020. https://cepr.net/wp-content/uploads/2020/04/2020-04-Frontline-Workers.pdf

[5] Bureau of Labor Statistics. Economic news release: job flexibilities and work schedules—2017–2018 data from the American Time Use Survey. Washington, DC: US Department of Labor, Bureau of Labor Statistics; 2019. https://www.bls.gov/news.release/flex2.htm

[6] Azar KMJ, Shen Z, Romanelli RJ, Disparities in outcomes among COVID-19 patients in a large health care system in California. Health Aff (Millwood) 2020;39:1253–62. 10.1377/hlthaff.2020.00598 32437224

[7] Tenforde MW, Billig Rose E, Lindsell CJ, ; CDC COVID-19 Response Team. Characteristics of adult outpatients and inpatients with COVID-19—11 academic medical centers, United States, March–May 2020. MMWR Morb Mortal Wkly Rep 2020;69:841–6. 10.15585/mmwr.mm6926e3 32614810 PMC7332092

